# Predictors of galcanezumab response in a real-world study of Korean patients with migraine

**DOI:** 10.1038/s41598-023-42110-4

**Published:** 2023-09-08

**Authors:** Seung Ae Kim, Hyemin Jang, Mi Ji Lee

**Affiliations:** 1 Department of Neurology, Seoul National University Hospital, Seoul, South Korea; 2 Seoul National University College of Medicine, Seoul, South Korea; 3 Department of Neurology, Samsung Medical Center, Sungkyunkwan University School of Medicine, Seoul, South Korea; 4 Alzheimer’s Disease Convergence Research Center, Samsung Medical Center, Seoul, South Korea

**Keywords:** Neurology, Headache

## Abstract

To assess factors associated with galcanezumab response in a real-world study of Korean patients with migraine. Predictors of the efficacy of monoclonal antibodies targeting calcitonin gene-related peptide (CGRP) or its receptor (anti-CGRP(-R) mAb) have been rarely investigated in Asians. We prospectively recruited and followed up patients with migraine who received monthly galcanezumab treatment in a single university hospital from June 2020 to October 2021. We defined the treatment response with ≥ 50% reduction in moderate/severe headache days in the 3rd month of treatment compared to baseline. Responders and non-responders were compared in terms of demographics, disease characteristics and severity, and previous response to migraine prophylactic treatments. Potential predictors of anti-CGRP(-R) mAb response were tested by using the univariable and multivariable logistic regression analyses. Among 104 patients (81.7% female; mean age 42.0 ± 13.02; 76.9% chronic migraine; and 45.5% medication overuse headache) included, 58 (55.7%) were responders. Non-responders had more chronic migraine, medication overuse headache, monthly headache days, days with acute medication, and daily headaches (i.e. chronic migraine persisting everyday without remission). The multivariable logistic analysis showed chronic migraine (OR 0.05 [95% CI 0.00–0.82], *p* = 0.036) and the number of previously failed preventive medication classes (OR 0.55 [95% CI 0.33–0.92], *p* = 0.024] were independently associated with treatment response. Chronic migraine and multiple failures from preventive medication are associated with poor galcanezumab response. Further studies are needed to investigate if earlier treatment before disease chronification or multiple failures may lead to a greater therapeutic gain from anti-CGRP(-R) mAb treatment.

## Introduction

Migraine is a global disease which is a leading cause of disability among adults of the most productive ages in their lives^1^. Oral preventive medications for migraine have been unsatisfactory to a considerable patient population due to low efficacy, intolerability, and low adherence rates^2^. Monoclonal antibodies targeting calcitonin gene-related peptide (CGRP) or its receptor [anti-CGRP(-R) mAbs] are emerging prophylactic treatment for migraine, of which four drugs (Galcanezumab, Fremanezumab, Erenumab and Eptinezumab) are FDA-approved and now considered as an important part of migraine treatment^3, 4^.Several randomized clinical trials have shown the efficacy and tolerability of anti-CGRP(-R) mAbs in patients with episodic migraine (EM), those with chronic migraine (CM), and those who were unresponsive to multiple migraine prophylactic medications^5–8^. However, the treatment outcome is not invariably excellent, and some proportion (e.g. for galcanezumab: 37.7–40.7% in EM, 70.4–81.3% in CM) of patients are considered as non-responders^5–17^.

There are several real-world studies of anti-CGRP(-R) mAbs currently available^18–36^. Further studies evaluated predictors of treatment response, and headache intensity, number of failed preventives, medication overuse, triptan response, unilateral pain, unilateral cranial autonomic symptoms, allodynia, age and baseline migraine days were reported to be associated with anti-CGRP(-R) mAb response^18, 21, 22, 27^. However, most of the studies are regarding erenumab in Europeans or North Americans as erenumab was the first anti-CGRP(-R) mAb approved in Europe and the United States. Real-world studies on predictors of response to anti-CGRP(-R) mAb other than erenumab are relatively scarce, and almost no such studies have been conducted outside of Europe or United States. In June 2019, Galcanezumab was approved in South Korea, which was the first anti-CGRP(-R) mAb marketing in Asia. Our group recently reported the real-world study of galcanezumab^37^. In the current study, we expanded the sample size and aimed to evaluate the predictors of galcanezumab response in our real-world data of Korean patients with migraine.

## Methods

### Participants

We prospectively recruited and followed up Korean patients with migraine from June 2020 to October 2021 who started monthly galcanezumab treatment and were registered in the prospective headache registry at the Samsung Medical Center headache clinic. Patients with other concomitant primary secondary headache disorders were excluded. The diagnosis of migraine, CM, and medication-overuse headache (MOH) was made by a headache specialist (MJL) according to the International Classification of Headache Disorders (ICHD-3)^38^. The Interstitial Review Board of the Samsung Medical Center approved this study, and all the participants gave a written informed consent.

### Evaluations

We collected baseline demographics, age at migraine onset, disease duration, headache phenotypes (unilaterality, nausea or vomiting, and photophobia and phonophobia), psychiatric comorbidities (depression and anxiety), migraine subtype (with vs. without aura), CM, MOH, baseline number of headache days, baseline number of days with moderate or severe headache (moderate/severe headache days), baseline number of days of acute medication, existence of pain-free day in the baseline month, previous response to triptans, number of previous failed medication classes, and the number of current oral prophylactic medication classes. The baseline numbers of headache days, moderate/severe headache days, and days of acute medication use were based on headache diaries in the 30 consecutive days before treatment. Anxiety and depression were defined as the Korean version of the Generalized Anxiety Disorder-7 score of ≥ 5 points and the Patient Health Questionnaire-9 score of ≥ 8 points^39^, respectively. Triptan response was defined as a positive response to the global assessment of relief in the Korean version of Migraine-ACT^40^. All the items were collected before the galcanezumab administration.

### Treatment protocol

Patients were administered with galcanezumab subcutaneously. In the first month, 240 mg was loaded and 120 mg was thereafter injected for the following two consecutive months. Other oral preventive medications, except botulinum toxin A (BTX), were allowed with stable doses. Acute headache medications were also allowed, with the same pattern and doses that the patients had used.

### Outcome

All the patients were prospectively followed up with the structured questionnaires regarding the treatment adherence, headache days, moderate/severe headache days, and days of acute medications of the last month. We defined the treatment response as ≥ 50% reduction in the number of moderate/severe headache days at the 3rd month (30 consecutive days) of treatment compared with baseline month^11, 41^.

### Statistical analysis

Depending on the their type and distribution, data are shown in numbers (percentages), mean (standard deviations), or median (interquartile range). SPSS version 22.0 software (SPSS Inc., Chicago, IL, USA) was used for the statistical analysis. The chi-square and Fisher's tests were used to compare categorical variables between responders and non-responders. Continuous variables were compared between groups using the student t-test or Mann–Whitney test according to the data distribution. Because factors were correlated with each other to a variable degree, the backward stepwise method was used to select variables for the multivariable logistic regression analyses. A two-sided *p*-value of < 0.5 was considered statistically significant.

### Ethics approval and consent to participate

The study protocol was approved by the institutional review board of each participating hospital in accordance with the 1964 Helsinki declaration, and written informed consent was obtained from all patients prior to recruitment.

## Results

### Demographics and characteristics of groups

A total of 104 patients (81.7% female, mean age 42.0 ± 13.02 years) completed evaluation and were included in the analysis. Demographics and characteristics of included patients are summarized in Table 1. Of included patients, 80 (76.9%) had chronic migraine and 46 (45.5%) had medication overuse headache. Patients had headache days of 21 (IQR 15–30), moderate/severe headache days of 10 (6–15), days of acute medication of 10 (5–15) in the baseline month. Most patients had failed two or more migraine prophylactic medication classes, and about half of patients (45.2%) failed 4 or 5 classes before enrollment.

**Table 1:** Demographics and characteristics of patients.

Female sex	85 (81.7%)
Age	42.0 ± 13.02
Disease duration	13.9 ± 12.21
Migraine with aura	17 (16.3%)
Chronic migraine	80 (76.9%)
Medication-overuse headache	46 (45.5%)
Unilaterality	51 (54.8%)
Nausea/vomiting	60 (63.8%)
Photophobia/phonophobia	66 (69.5%)
Baseline headache days/m	21 (15–30)
Baseline moderate/severe headache days/m	10 (6–15)
Baseline days of acute medication/m	10 (5–15)
Presence of pain-free days in the baseline month	59 (57.8%)
Depression	25 (40.3%)
Anxiety	30 (48.4%)
Triptan response	69 (73.4%)
Previously failed prophylactics class (n)	
0	11 (10.6%)
1	7 (6.7%)
2	16 (15.4%)
3	23 (22.1%)
4	23 (22.1%)
5	24 (23.1%)
Current preventive medication (n)	
0	34 (33.0%)
1	15 (14.6%)
2	33 (32.0%)
3	19 (18.4%)
4	1 (1.0%)
5	1 (1.0%)

### Characteristics of responders and non-responders

Of 104 patients included in this study, 58 (55.7%) were 50% responders. The baseline characteristics were compared between responders and non-responders (Table 2). Non-responders had more CM, MOH, headache days, and days of acute medication, and less prevalence of pain-free day in the baseline period (Table 2). The number of previously failed prophylactic medication classes was greater in non-responders than in responders (median 4 [IQR 3–5] vs. 3 [IQR 2–4], respectively), but it did not reach the statistical difference (*p* = 0.064).

**Table 2:** Comparison of demographics and disease characteristics between responders and non-responders.

	Non-responder (n = 46)	Responder (n = 58)	*P*-value
Age	42 ± 12.26	41.9 ± 13.69	0.973
Female sex	39 (84.8%)	46 (79.3%)	0.473
Disease duration	10.5 (2–20)	13 (5–25)	0.445
Migraine with aura	9 (19.6%)	8 (13.8%)	0.429
Chronic Migraine	42 (91.3%)	38 (65.5%)	0.002
Medication-overuse headache	25 (58.1%)	21 (36.2%)	0.029
Unilaterality	25 (58.1%)	26 (52.0%)	0.553
Nausea/vomiting	31 (72.1%)	29 (56.9%)	0.126
photophobia/phonophobia	28 (65.1%)	38 (73.1%)	0.402
Baseline headache days/m	30 (19–30)	16 (12–30)	0.008
Baseline moderate/severe headache days/m	12 (5.5–15.5)	10 (5–15)	0.367
Baseline days of acute medication/m	12 (5–30)	7 (4–10)	0.043
Existence of pain-free days/m	21 (45.7%)	38 (67.9%)	0.024
Depression	14 (50.0%)	11 (32.4%)	0.159
Anxiety	16 (57.1%)	14 (41.2%)	0.211
Triptan response	29 (65.9%)	40 (80.0%)	0.123
Previously failed prophylactic medication class (n)	4 (3–5)	3 (2–4)	0.064
Current prophylactic medication class(n)	34 (73.9%)	36 (62.1%)	0.201

### Factors associated with galcanezumab response

Univariable analyses showed CM, MOH, higher number of baseline headache days, greater acute medication use, and higher number of previously failed medication classes were associated with galcanezumab response (Table 3). Variables were tested and selected by using the backward stepwise method for the multivariable logistic regression analyses. The final model showed chronic migraine (OR 0.09 [95% CI 0.01–0.97], *p* = 0.047) and the number of previously failed preventive medication classes (OR 0.55 [95% CI 0.32–0.92], p = 0.022] were independently associated with galcanezumab response (Table 3).

**Table 3:** Factors associated with galcanezumab response.

	Univariable OR (95% CI)	*P* value	Multivariable OR (95% CI)	*P* value
Age	1.00 (0.97–1.03)	0.973		
Female sex	1.45 (0.52–4.05)	0.475		
Disease duration	1.02 (0.98–1.06)	0.330		
Migraine with aura	0.59 (0.20–1.68)	0.321		
Chronic migraine	0.18 (0.06–0.58)	0.004	0.09 (0.01–0.97)	0.047
Medication-overuse headache	0.41 (0.18–0.92)	0.030		
Unilaterality	0.78 (0.34–1.77)	0.553		
Nausea/vomiting	0.51 (0.21–1.21)	0.128		
photophobia/phonophobia	1.45 (0.61–3.49)	0.403		
Baseline headache days/m	0.94 (0.89–0.98)	0.010		
Baseline moderate/severe headache days/m	1.02 (0.97–1.07)	0.364		
Baseline days of acute medication/m	0.95 (0.90–1.00)	0.048		
Existence of pain-free days/m	2.51 (1.12–5.63)	0.025		
Depression	0.48 (0.17–1.34)	0.161		
Anxiety	0.53 (0.19–1.45)	0.213		
Triptan response	2.07 (0.81–5.25)	0.126		
Previously failed prophylactic medication class (n)	0.67 (0.48–0.94)	0.021	0.55 (0.32–0.92)	0.022
Current prophylactic medication class (n)	0.81 (0.62–1.04)	0.097		

## Discussion

Main findings of this study are as follows: (1) galcanezumab response rates were higher than reported in clinical trials in this real-world study of Korean patients with migraine and (2) chronic migraine and multiple failures from preventive medication were associated with poor galcanezumab response.

In this study, a high response rate (55.7% overall, 83.3% in EM, and 47.5% in CM) to 3-month treatment of galcanezumab was observed. Participants of this study overlapped with those included in our recent study^37^ where the response rates were 54.5% and 41.5% for EM and CM, respectively. Nevertheless, this study showed a higher response rate particularly in EM. This discrepancy might have been attributed to we extended the study period and recruited more patients after the initial study that included patients with refractory migraine who had waited the approval of anti-CGRP(-R) mAb received galcanezumab treatment soon after approval. Afterwards, galcanezumab became more popular medication due to increased awareness among patients, and more patients with less disease severity and previous failures were included in this study. Galcanezumab was not reimbursed by national health insurance system during the study period in Korea, which means the galcanezumab use was not controlled by government and could be a first-line treatment if patients wanted. Although the high cost could have limited the access to the drug, we were able to enroll patients with a wide spectrum of disease severity. Our current study might be closer to a real response to galcanezumab, while our previous study reflects a response rate of galcanezumab in highly treatment-resistant migraine. Given the overarching importance of migraine as a substantial public health issue^42–46^, the integration of high-cost innovative medications such as galcanezumab while adhering to safety recommendations^47^, assumes a critical role in influencing treatment strategies. This contribution holds the potential to enhance migraine patient outcomes and elevate overall quality of life.

In our study, CM was independently associated with poor galcanezumab response. This reflects that disease severity and refractoriness were key determinants of galcanezumab response. Clinical trials of anti-CGRP(-R) mAbs yielded overall lower 50% responder rates in CM^7, 15–17^ than in EM^5, 6, 10, 13^, and similarly, lower response rates were observed in trials of patients with multiple previous treatment failures^8, 9, 12, 14^ than in trials where patients with multiple treatment failures were excluded^5–7, 10, 13, 15–17^. Real-world studies that examined the difference between EM and CM are relatively scarce, as most studies included only CM patients^18, 20–22, 24, 25, 28, 29, 33, 34, 36, 48, 49^. Schoenen et al. compared the 50% responder rates between EM and CM patients in their study of 1-year erenumab program and reported higher a response rate in EM compared to CM^25^. Studies of CM examined the disease duration after chronification, and most studies reported negative results^18, 20–22, 24, 33, 34, 49^. This may imply the disease characteristics of CM, at least in terms of treatment response, is not merely attributed to the temporal chronicity of the disease. Rather, patients with CM may have either genetic predisposition or biological properties that utilizes neuropeptides, neurotransmitters, or intracellular mechanisms different to the CGRP-directed pathway.

Higher number of previous migraine prophylactic medications was also significantly associated with the poor galcanezumab response in our study. This was investigated in several other studies, and the results were highly controversial. There are three positive studies on the association between the number of previously failed medications and anti-CGRP(-R) mAb response^20, 22, 25^, whereas several other studies reported a negative association^18, 21, 24, 27, 29, 33–36, 48–50^. The discrepancy might have come from that most studies recruited refractory chronic migraine, whereas our study included treatment-naïve patients, which could enable the comparison across the whole spectrum of treatment experience. Our study supports the view that the number of failed migraine prophylactic medications is a significant predictor of galcanezumab. It can be hypothesized that multiple failures from other migraine prophylactic medications would reflect a biological state where pain modulation pathway is impaired^51–53^. This emphasizes the need for early consideration of treatment options, even before trying multiple unsuccessful approaches, to improve treatment results. In essence, evaluating Galcanezumab's effectiveness earlier could offer more advantages before patients undergo a series of ineffective preventive methods. In line with the 2022 EHF guidelines^54^, which suggest the use of anti-CGRP monoclonal antibodies as a primary option for migraine management, the consideration of initiating such treatment for all migraine patients, regardless of their subtype or treatment history, holds potential for gaining valuable insights into individual responses and optimizing treatment strategies. Further studies are needed to investigate if earlier treatment with anti-CGRP(-R) mAbs before multiple trials of oral prophylactic medications would yield a higher response rate and eventually lead to a lower prevalence of “difficult-to-treat” migraine.

Our study has some strengths. This was the first real-world responder analysis purely dealing with galcanezumab. A prospective design and a relatively wide spectrum of disease severity are considered as other strengths as discussed earlier. To our best knowledge, there is currently no published series on anti-CGRP(-R) mAb response predictors in the Asian population. Our study is not without limitations. First, this was a monoethnic study from a single center. Further studies from other Asian regions are needed before generalizing our study findings. Second, we were unable to measure the serum concentration of anti-CGRP(-R), thus we could not conclude if the non-response was potentially mediated by pharmacodynamic or pharmacokinetic differences.

## Conclusion

In conclusion, this study showed that galcanezumab is an effective preventive treatment in migraine in Korean population. CM and higher numbers of previous preventive medication are associated with poor galcanezumab response.

## Data Availability

Mi ji Lee* should be contacted if there are any inquiries about the data. All data and materials generated in this study will be available upon request under the approval from the institutional review board of Samsung Medical Center.
